# Myocarditis in Cats with Feline Infectious Peritonitis Can Be Cured with GS-441524 and Symptomatic Cardiovascular Treatment

**DOI:** 10.3390/ani15111660

**Published:** 2025-06-04

**Authors:** Katharina Buchta, Jana Friederich, Anna-Maria Zuzzi-Krebitz, Jessica Schöbel, Jenny Eberhard, Katharina Zwicklbauer, Andrea M. Spiri, Regina Hofmann-Lehmann, Katrin Hartmann, Gerhard Wess

**Affiliations:** 1 LMU Small Animal Clinic, Centre for Clinical Veterinary Medicine, LMU Munich, 80539 Munich, Germany; jana.friederich@lmu.de (J.F.); fipmunich@gmail.com (A.-M.Z.-K.); j.schoebel@medizinische-kleintierklinik.de (J.S.); eberhard@tierklinik-elversberg.de (J.E.); k.zwicklbauer@medizinische-kleintierklinik.de (K.Z.); hartmann@lmu.de (K.H.); gwess@lmu.de (G.W.); 2 Clinical Laboratory, Department of Clinical Diagnostics and Services, Center for Clinical Studies, Vetsuisse Faculty, University of Zurich, CH-8057 Zurich, Switzerland; aspiri@vetclinics.uzh.ch (A.M.S.); regina.hofmann-lehmann@uzh.ch (R.H.-L.)

**Keywords:** feline coronavirus, FIP, systolic dysfunction, arrhythmia, antivirals

## Abstract

Feline infectious peritonitis (FIP) caused by feline coronavirus (FCoV) is a fatal disease in cats characterized by variable manifestations. Myocarditis represents a less commonly described pathology within the clinical spectrum of FIP. No research has described the successful treatment of FIP-induced myocarditis. In this study, 40 cats were included and treated with 15 mg/kg of GS-441524 every 24 h orally. All cats were diagnosed with FIP by reverse transcription quantitative PCR in effusion in combination with typical clinical and laboratory changes. Echocardiography was performed in all cats, and myocarditis was suspected in 4/40 cats. Equivocal wall thickness was diagnosed in 2/4 cats, while systolic dysfunction with biatrial dilation was diagnosed in the other 2/4 cats. One cat also presented with ventricular ectopy. A severe increase in cardiac troponin I was seen in all four cats (median 1.82 ng/mL (1.20–5.84 ng/mL)). Cardiac dimensions and electrocardiographic abnormalities completely normalized in all four cats during treatment with GS-441524 and remained stable after treatment discontinuation during a one-year follow-up period. Myocarditis can be a clinical feature of FIP and present with different cardiologic manifestations. FIP-induced myocarditis can be cured with GS-441524 in combination with symptomatic cardiovascular treatment including pimobendan, clopidogrel, furosemide, or atenolol, depending on the clinical presentation.

## Simple Summary

Feline infectious peritonitis (FIP) is a fatal disease in cats and can present in various manifestations. Myocarditis is a, so far, rarely described pathology within the clinical spectrum of FIP. In the present study, suspicion of myocarditis was raised in 4/40 cats that presented with different cardiac manifestations. These four cats completely recovered during treatment with GS-441524 in combination with cardiovascular therapy and remained stable during a one-year-follow-up. Myocarditis can be an important feature in cats with FIP and should therefore be closely monitored in these cats.

## 1. Introduction

Feline infectious peritonitis (FIP) caused by feline coronavirus (FCoV) is one of the most common infectious diseases in cats with fatal consequences if untreated [1]. The antiviral nucleoside analog GS-441524 has demonstrated high efficacy against FIP [2,3,4,5,6,7,8,9,10,11,12,13,14,15,16,17,18]. Cats with FIP can develop body cavity effusions, pyogranulomatous lesions in different organs including brain and eyes [19], as well as less common signs, such as dermatological manifestations [20,21,22]. Recently, cases of myocardial lesions [23,24] or myocarditis have also been described [25,26,27,28,29].

Myocarditis is characterized by the infiltration of inflammatory cells into the myocardium, often accompanied by degenerative alterations of cardiomyocytes. Myocarditis can present with different clinical signs; observed changes can include systolic and/or diastolic dysfunction, ventricular wall thickening and abnormal wall motion, pericardial effusion, and electrocardiographic changes [30].

Inflammation of the myocardium can arise from both infectious and non-infectious causes. Examples of infectious agents in cats include bacteria, such as *Streptococcus suis* [31], *Bartonella henselae* [32,33] causing (pyo-) granulomatous myocarditis, or *Salmonella typhimurium* [34] resulting in bacteremia-associated myocarditis. Myocarditis can also be caused by parasites, such as *Hepatozoon silvestris* [35], leading to lymphoplasmacytic and histiocytic myocarditis, as well as *Sarcocystis felis* [36] and *Toxoplasma gondii* [37,38]. Viruses other than FCoV have rarely been associated with myocarditis in cats. One study identified feline immunodeficiency virus (FIV) antigens in inflammatory cells, e.g., T-lymphocytes and macrophages, in the heart of cats with myocarditis and hypertrophic cardiomyopathy (HCM) phenotype. However, a definitive association between FIV and the development of myocarditis was not confirmed [39]. One case report described an association of severe acute respiratory syndrome coronavirus type 2 (SARS-CoV-2) infection with heart failure in a cat [40]. However, a myocardial biopsy was not performed, and it remains uncertain whether the virus directly induced myocarditis or whether it was secondary to a multisystem inflammatory syndrome, as described in humans [41]. In addition, FCoV has been detected by immunohistochemistry (IHC) in the myocardium in cats with FIP in necropsy [25,26]. In two cats that presented with a dilated cardiomyopathy (DCM) phenotype, necropsy revealed diffuse granulomatous inflammation in the heart, which led to the retrospective diagnosis of FIP [27].

Myocarditis in cats with FIP has not yet been prospectively described in living cats. Additionally, nothing is known about the development of myocarditis during the antiviral treatment of FIP. Therefore, the aims of this study were to describe the manifestation of FIP-associated myocarditis in a clinical setting and evaluate the outcome in cats with FIP-associated myocarditis treated with GS-441524 in combination with cardiovascular treatment.

## 2. Materials and Methods

### 2.1. Study Population and Protocol

Forty cats were originally included in a previously published prospective study by Zuzzi-Krebitz and colleagues (2024) [5]. In four cats, the suspicion of myocarditis was raised. Inclusion criteria for study participation in the previously published study were the presence of puncturable effusion, a negative result for FIV antibodies and feline leukemia virus (FeLV) antigens, and a diagnosis of FIP. The diagnosis of FIP was made based on a combination of clinical and laboratory parameters typically altered in cats with FIP, along with a positive FCoV reverse transcription quantitative PCR (RT-qPCR) from effusion. Clinical signs typical for FIP included lethargy, fever, hyporexia, and pleural/abdominal effusion. Laboratory findings included aregenerative anemia, lymphopenia, hyperbilirubinemia, hypalbuminemia, decreased albumin/globulin ratio (A/G), and elevated acute phase proteins [19,42,43] (Table 1).

All cats were treated with GS-441524 (15 mg/kg, every 24 h (q24h), per os (PO)). They were hospitalized in the clinic for seven days and underwent a detailed physical examination as well as laboratory analyses and abdominal ultrasonography. In addition, cardiac evaluations including electrocardiography (ECG) and echocardiography were performed in all cats before the initiation of GS-441524 treatment. After discharge from the hospital (LMU small animal clinic, Munich, Germany) on day 7, cats were treated at home and were not allowed to leave the house during the antiviral treatment period. All cats received a commercial diet. They were monitored on days 14, 28, 42, 56, 84, 168, 252, and 365 after treatment initiation. At these timepoints, all cats underwent a physical examination, abdominal ultrasonography and cardiac assessment (days 42, 84, 168, and 365). Laboratory analyses, including complete blood cell count (CBC) and serum biochemistry, were performed. Viral loads in blood, effusion, and feces were determined by RT-qPCR. Details on the evaluation of laboratory parameters and FCoV RT-qPCR are described in the original study [5].

This study complied with the German guidelines for prospective studies and was approved by the Government of Upper Bavaria (reference number 55.2-2532.Vet_02-20-52) and by the ethical committee of the Centre for Clinical Veterinary Medicine of LMU Munich (reference number 261-19-03-2021). Owners gave their written informed consent for their cats to participate in the study.

### 2.2. Cardiac Examination

All 40 originally included cats [5] received a detailed cardiac examination, including the precise auscultation of the heart, ECG, and echocardiography. In cats with suspicion of myocarditis, cardiac troponin I (cTnI) was measured in fresh and cooled serum (reference interval (RI): <0.06 ng/mL) by chemiluminescence using Siemens^®^ ADVIA Centaur XP (IDEXX Laboratories, Kornwestheim, Germany).

A 5 min 6-lead ECG (EKG 2000, Eickemeyer Medizintechnik, Tuttlingen, Germany) was recorded in right lateral recumbency without sedation. The underlying rhythm was determined, and the presence, number, and coupling interval of ventricular (VPCs) and atrial premature contractions (APCs) were documented. Additionally, signs of complexity (couplets and triplets) and malignancy (R-on-T phenomenon and ventricular tachycardia (VT)) or the presence of conduction abnormalities like atrioventricular blocks or bundle branch blocks were recorded.

Echocardiography (12–4 MHz transducer, Epiq 7, Philips Healthcare, Hamburg, Germany) was performed in right and left lateral recumbency without sedation, according to the published recommendations [44]. Interventricular septal (IVSd) and left ventricular posterior wall (LVPWd) thickness in end-diastole was measured in 2D using the leading edge-to-leading edge method. Left ventricular (LV) wall thickness ≥5 mm and <6 mm was considered equivocal, and hypertrophy was defined as IVSd and/or LVPWd ≥6 mm [45,46]. In cats >6 kg, weight-dependent reference intervals were used [47]. The left atrial-to-aortic root ratio (LA/Ao) was measured in right parasternal short-axis at end-systole as previously described [48]. The left atrial diameter (LAD) was measured in right parasternal long-axis optimized for the left atrium one frame before the mitral valve opening, as recommended [49]. Left atrial enlargement was defined as LA/Ao ≥1.6 and LAD ≥16 mm [49,50]. The right atrial diameter (RAD) was measured similarly to the LAD, and right atrial enlargement was diagnosed with RAD ≥12 mm [51]. Reduced systolic function was diagnosed with increased LV dimensions, especially in end-systole (LVIDs), and subsequently in end-diastole (LVIDd) according to breed-specific reference intervals [47]. In cats with suspected systolic dysfunction, the left ventricular global longitudinal strain (GLS) was measured using speckle-tracking echocardiography on an external work station (TOMTEC Imaging Systems GmbH, Unterschleißheim, Germany), as previously described [52]. According to previously published reference intervals for cats, reduced systolic function was defined as a GLS less negative than −21.18% [53].

Congestive heart failure (CHF) was confirmed when there was radiographic evidence of pulmonary edema, ultrasonographic detection of pleural and/or pericardial effusion, and associated clinical signs which resolved after the administration of diuretics [54].

### 2.3. Diagnosis of FIP-Induced Myocarditis

The modified Duke criteria proposed for the diagnosis of infectious endocarditis in humans [55], dogs [56], and cats [57] that have been adjusted for the diagnosis of myocarditis in dogs [58] were applied. According to these classifications, major and minor criteria have been proposed. The major criteria included cTnI >1.0 ng/mL and/or the detection of an infectious organism using a culture of blood/body fluid or PCR/RT-PCR. The minor criteria included a fever, a new or worsening heart murmur, ventricular arrhythmias, decreased left ventricular systolic function, heteroechogenicity of the left ventricular myocardium, pericardial effusion, and laboratory changes such as inflammatory leukogram, anemia, thrombocytopenia, and hypoalbuminemia. A high suspicion of myocarditis was raised if either two major criteria or one major and three minor criteria were fulfilled [58].

## 3. Results

Four cats were diagnosed with myocarditis (4/40; 10%) based on the fulfillment of two major criteria (cTnI >1.0 ng/mL and diagnosis of FIP) and additionally between one and two minor criteria for the diagnosis of myocarditis, as previously described [58]. All four cats were treated with GS-441524 at a dosage of 15 mg/kg q24h PO for 84 days. Additional treatment can be found in Table 2.

### 3.1. Cardiac Findings

Cardiac auscultation was unremarkable in two cats (cats 38 and 39), one cat presented with gallop rhythm (cat 24), and one cat was arrhythmic with pulse deficits (cat 40). A severe increase in cTnI levels was seen in all four cats, with a median of 1.82 ng/mL (1.20–5.84 ng/mL) (RI < 0.06 ng/mL).

Two cats (cats 38 and 40) presented with equivocal cardiac wall thickness and heteroechogenicity of the left ventricular myocardium. The left atrial size was normal in both cats (Table 3). Cat 38 presented with equivocal cardiac dimensions and heterogenous echogenicity of the LV walls, which is considered abnormal in a young cat. In combination with severely elevated cTnI (1.37 ng/mL), acute myocardial damage was suspected and the suspicion of myocarditis was raised.

**Figure 1:**
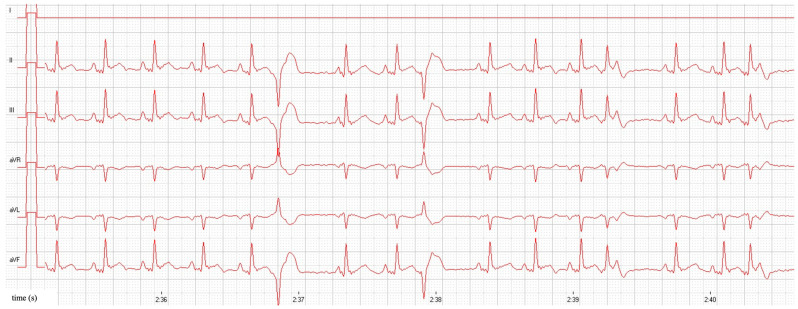
Atrial and ventricular premature contractions in a cat with equivocal cardiac dimensions (cat 40). Before treatment, sinus rhythm with a heart rate of 166 bpm, single VPCs with a coupling interval of 333 bpm, and single APCs with a coupling interval of 320 bpm can be seen. Amplitude 10 mm/mV, paper speed 50 mm/s. APC = atrial premature contraction; VPC = ventricular premature contraction.

Cat 40 also had 50 single VPCs in five minutes with a maximum coupling interval of 333 bpm, 28 single APCs with a maximum coupling interval of 320 bpm, and 7 fusion beats (Figure 1). Based on these findings, atenolol (1.5 mg/kg, q12h, PO) was started in cat 40. No further cardiac treatment was otherwise indicated in those two cats.

Cat 24 presented with systolic dysfunction based on increased LVIDs above breed-specific reference intervals [47] and reduced LV GLS (−10.19%) (Table 4). The left and right ventricle were affected, and the cat showed biatrial enlargement. Due to the presence of pulmonary edema, the cat was considered to be in CHF and treatment with furosemide (1.25 mg/kg, q12h, PO), pimobendan (0.3 mg/kg, q12h, PO), and rivaroxaban (0.6 mg/kg, q24h, PO) was started (Table 2). Prior to the start of the study, the cat was additionally diagnosed with uveitis and chorioretinitis caused by FIP and was therefore treated with prednisolone acetate eye drops. These eye drops were substituted with ketorolac-trometamol eye drops on day 12. This change in eye drop formulation was necessary due to supply shortages, which made the originally administered product temporarily unavailable. On the same day, the general condition of this cat deteriorated and the cat showed symptoms of fever, vomiting, and erythema cutis. At this time, sinus tachycardia with an instantaneous rate of 220 bpm, which was interrupted by a single episode of VT with an instantaneous rate of 340 bpm, was noted on monitor observation. The VT converted into sinus rhythm after approximately three seconds without medical intervention. The ECG of the cat was closely monitored for the next days; the VT never recurred, and only rare isolated VPCs that did not require any treatment could be detected. Rivaroxaban had to be discontinued on the same day due to petechiae and pronounced thrombocytopenia (68.00 ×10^9^/L; RI: 180–550) of unknown origin but was restarted once the platelet count returned to the reference interval on day 19. As these abnormalities improved within a few days after the discontinuation of the ketorolac-trometamol eye drops, drug reaction was suspected.

Cat 39 also presented with systolic dysfunction based on increased LVIDs above breed-specific reference intervals [47] and reduced LV GLS (−16.22%) (Table 4). The left and right ventricle were affected, and the cat showed biatrial enlargement. The cat also showed mild pericardial effusion, which was thought to be secondary to FIP and not a sign of congestion in this cat. The cat was started on pimobendan (0.24 mg/kg, q12h, PO) and clopidogrel (18.75 mg/cat, q24h, PO). Diuretics were not considered necessary (Table 2). The echocardiographic data of all four cats can be seen in Table 3 and Table 4.

### 3.2. Outcome

**Figure 2:**
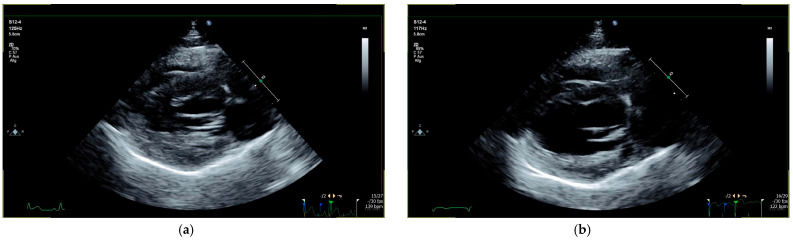
Echocardiographic follow-up in a cat with equivocal cardiac dimensions (cat 40): (**a**) before treatment, cat 40 presented with equivocal left ventricular wall thickness (IVSd 5.1 mm and LVPWd 5.6 mm) and VPCs at the initial examination. (**b**) Day 42 after treatment initiation, cardiac dimensions completely normalized (IVSd 4.9 mm and LVPWd 4.8 mm). IVSd = interventricular septum thickness at end-diastole; LVPWd = left ventricular posterior wall thickness at end-diastole; VPC = ventricular premature contraction.

**Figure 3:**
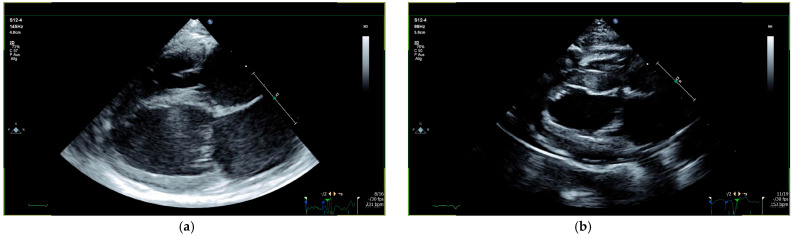
Echocardiographic follow-up in a cat with systolic dysfunction (cat 24): (**a**) before treatment, cat 24 presented with systolic dysfunction (LVIDs 13.5 mm and GLS −10.19%) and biatrial dilation (LA/Ao 2.43, LAD 17 mm, RAD 13.1 mm) at the initial examination; (**b**) day 84 after treatment initiation, systolic function (LVIDs 10.6 mm and GLS −28.29%) and atrial dimensions (LA/Ao 1.44, LAD 14 mm, RAD 10.9 mm) completely normalized. GLS = global longitudinal strain; LA/Ao = left atrial-to-aortic root ratio; LAD = left atrial diameter; LVIDs = left ventricular internal diameter at end-systole; RAD = right atrial diameter.

All four cats had already presented with cardiac changes prior to treatment, but in three cats, the cardiac situation deteriorated within the first days after treatment initiation. However, during the treatment with GS-441524, all four cats recovered completely from their suspected myocarditis based on the normalization of the cardiac dimensions. Except for cat 24, abnormal cardiac parameters normalized in all cats by day 42, and all cardiac medications could be stopped in those three cats on the same day. Cat 24 still had signs of systolic dysfunction on day 42, and cardiovascular treatment (pimobendan, clopidogrel, and furosemide) was continued until day 84 when the cardiac dimensions normalized and the cardiovascular treatment could also be discontinued. The arrhythmias in cat 40 had already resolved by day 14 (based on 10 min ECG recording). Echocardiography and ECG were unremarkable in all cats during the follow-up examinations on days 168, 252, and 365 after treatment initiation. Echocardiographic follow-ups are demonstrated in Figure 2 and Figure 3. All clinical and laboratory signs typical of FIP improved during antiviral treatment [5]; in most cats, the improvement of other clinical and laboratory parameters (normalization by day 28–42 at the latest), such as the Karnofsky’s score, hematocrit, bilirubin concentration, and albumin concentration, occurred more rapidly than the recovery from FIP-induced myocarditis (Table 1).

## 4. Discussion

The results of this study demonstrate that myocarditis can be an important feature of FIP and can be effectively treated with an effective antiviral compound when combined with symptomatic treatment with cardiovascular drugs. The variable manifestation of myocarditis and a lack of validated diagnostic criteria complicates the diagnosis, but cardiac changes successfully resolve during treatment.

Myocarditis is a rare problem in cats, but a few reports about FIP-induced myocarditis exist [25,26,27,28,29]. The low number of case reports about FIP-associated myocarditis likely depends on their relatively low importance compared with other lesions caused by FIP. So far, diagnosis in those previously described cases was determined postmortem by histopathological examination and not by detailed cardiologic assessments of living cats. Two previous histopathology reports [25,26] identified pyogranulomatous infiltration in the myocardium, and IHC confirmed the presence of FCoV-positive macrophages. Multifocal areas of pyogranulomatous vasculitis were observed in these cats, were characterized by macrophages, neutrophils, and occasional lymphocytes, and were frequently centered around cores of lytic necrosis. The observed thickening of the myocardium was attributed to edema and the presence of inflammatory infiltrates, including lymphocytes, plasma cells, and FCoV-positive macrophages [25,26]. However, these cases only described changes in necropsy, and, so far, no detailed descriptions of FIP-associated myocarditis in cats in vivo and during their follow-up exist. There are also no reports available on the clinical or therapeutic management of cardiomyopathy in cats with FIP, likely because, until a few years ago, FIP was considered invariably fatal. Therefore, recent research focused primarily on the antivirals available for treatment rather than providing recommendations or guidelines for the supportive care of associated diseases such as cardiomyopathies. With the availability of efficient antiviral drugs, there is now an opportunity and necessity to investigate these additional treatments.

This study is the first describing the echocardiographic features of in vivo FIP-associated myocardial changes and follow-up examinations during antiviral treatment. In the described cats, either equivocal wall thickness or systolic dysfunction with biatrial enlargement were the most important findings, and both manifestations completely resolved with treatment with GS-441524. In severe cases, it should be considered that additionally supportive cardiovascular treatment is necessary.

Biatrial enlargement or hypertrophic phenotype have been previously described as “comorbidities” associated with FIP [3,13,26]. These findings suggest cardiac involvement in some cats with FIP. The presence of cardiac manifestations in 10% of the originally included cats suggests that FIP-induced myocarditis is probably more common than previously thought. Thus, it is important to consider myocarditis in cats with FIP, measure cTnI for the evaluation of cardiomyocyte damage, and potentially refer the cats for a detailed cardiac examination. Pleural effusion is not only a consequence of vasculitis caused by FIP but can also be a sign of heart failure due to myocarditis.

Endomyocardial biopsy is considered the gold standard for the in vivo diagnosis of myocarditis in humans [59]. However, since this procedure is highly invasive in cats [60], a suspected diagnosis often relies on historical and clinical data in combination with laboratory findings such as cTnI [61], ECG, and echocardiography [58,62]. The criteria proposed by Lakhdhir and colleagues (2020) for dogs [58] can be used as diagnostic tools to confirm the suspicion of myocarditis. In the present study, all four cats fulfilled two major criteria (cTnI > 1.0 ng/mL and diagnosis of FIP), as well as between one and two minor criteria, including decreased left ventricular systolic function (cats 24 and 39), pericardial effusion (cat 39), ventricular arrhythmias (cat 40), and the heteroechogenicity of the myocardium (cats 38 and 40). Certain laboratory changes, such as the elevation of acute phase proteins and fever can be related to FIP itself [19,42,43] and are therefore not indicative of myocarditis in cats with FIP.

In this study, NTproBNP levels were not measured, as cTnI is generally considered a more sensitive marker for myocardial damage, and it can be severely elevated in cases with myocarditis [63]. On the other hand, NTproBNP primarily reflects myocardial stretch or volume overload and has a strong correlation with the left atrial size. It is therefore suited to diagnose moderate to severe echocardiographic changes, but equivocal or mild disease might not be detected [64,65]. The in-house SNAP test provides a qualitative result (normal vs. abnormal) and might only be helpful in severe cases to distinguish cardiac-related respiratory distress from non-cardiac related disease [66]. Normal SNAP test results do not guarantee the absence of cardiomyopathy, especially in cases of mild heart disease [67]. It is therefore recommended to measure cTnI as a more appropriate biomarker for myocardial damage in combination with echocardiographic examination.

SARS-CoV-2 can cause myocarditis in humans [68] and is associated with an inflammatory reaction and cytokine storm [69]. Cardiac complications of coronavirus disease 2019 (COVID-19) include acute myocardial injury with increased cTnI levels, decreased ejection fraction, arrhythmias, thromboembolism, and pericarditis [70]. COVID-19-associated myocarditis is caused by direct cell injury and T-lymphocyte-mediated cytotoxicity, possibly being augmented by cytokine storm syndrome, especially by interleukin 6 (IL-6) [71,72]. Interestingly, SARS-CoV-2 has also been reported to cause myocarditis in cats [40,73,74], and further studies are needed to determine if FCoV infects the myocardium in a similar manner. In one study, it could be shown that cats with FIP display the increased transcription of inflammatory cytokines in the heart muscle and liver when compared to cats without FIP, although the increase was lower in the heart compared to the liver. This suggests a later and more reactive involvement of the myocardium in the disease process [75]. In the present study, cTnI was higher in the two cats with systolic dysfunction (2.27 and 5.84 ng/mL) compared to the two equivocal cats (1.37 and 1.20 ng/mL), and more pronounced cardiac damage as a result of the cytokine reaction may be a possible explanation for the different disease manifestations.

The use of the modified Duke’s criteria to diagnose myocarditis might be controversial in a multisystemic disease such as FIP. Without the use of endomyocardial biopsies, it remains unclear whether the virus itself and the associated inflammatory reaction or the massive cytokine response were responsible for myocarditis in the affected cats. However, in the present cases with a definitive diagnosis of FIP and the remission of myocarditis after treatment initiation, it is highly likely that the changes in the myocardium were caused by FIP.

All of the affected cats in this study exhibited cardiac changes prior to the initiation of treatment, but in three cats, the cardiologic situation deteriorated within the first days after treatment initiation. However, since the echocardiographic measurements and the general condition of the cats overall improved with GS-441524 treatment, it is strongly presumed that myocarditis is a consequence of FIP and not an adverse reaction to the drug. Thus, GS-441524 can be considered an effective and safe treatment for FIP-induced myocarditis. However, it is also important to mention that severe myocardial damage might not always be reversible with GS-441524 and supportive cardiovascular treatment. Furthermore, myocarditis should be recognized as a serious comorbidity in cats with FIP.

All cats received a uniform dosage of 15 mg/kg q24h PO, regardless of the presence of additional neurologic or ocular signs. In a previous publication by Krentz and colleagues (2021) [15], cats were treated with 5 mg/kg q24h PO GS-441524, increasing the dose to 10 mg/kg in cats with neurological or ocular involvement. However, the analysis of the multicomponent drug (Xraphconn^®^) used in this study revealed that the actual active GS-441524 content was more than twice the amount stated by the manufacturer (personal communication, J. Horak). Based on these findings, a dosage of 15 mg/kg was selected for all FIP manifestations. The four cats in this case series were treated for 84 days. They were randomly assigned to the long treatment group [5], and this decision was not based on the presence of cardiac manifestations.

Both myocarditis manifestations completely resolved with treatment with GS-441524. However, it could be argued that myocarditis resolved on its own or without causal treatment with GS-441524, as seen in stress-induced transient myocardial thickening (TMT) in cats [76]. Nevertheless, without GS-441524, the cats with FIP would have died from the disease itself and myocarditis would have had no chance to resolve on its own.

A limitation of this study was that cTnI was only measured in those of the 40 included cats in which myocarditis was suspected based on echocardiography, and cats with mild cardiac damage could have been missed. CTnI was not reassessed in cats with suspected myocarditis, and the reassessment relied solely on the improvement observed in cardiac dimensions. Further prospective studies with larger sample sizes should be performed in the future. As another limitation, only a high suspicion of myocarditis was raised and no definitive diagnosis via biopsy was made. Other reasons for myocarditis in cats, such as *Toxoplasma gondii* [37,38] or *Bartonella henselae* [32,33], were not excluded. However, due to the improvement of the general condition and cardiac changes during treatment with GS-441524, it was considered to be proven that myocarditis was FIP-induced.

## 5. Conclusions

This study demonstrates that myocarditis can be an associated feature of FIP and therefore should be considered as a potential complication. A thorough physical examination and cTnI testing are recommended in cats with FIP, and in cases of suspected myocarditis and/or clinical deterioration, a comprehensive cardiological assessment should be performed. FIP-associated myocarditis can effectively be cured with GS-441524 in combination with individually adapted cardiovascular treatment. This study also demonstrates that it is extremely important that FIP and all associated features are properly diagnosed and managed by veterinarians, ideally in veterinary hospitals with specialists who are able to manage complicated and critical cases.

## Figures and Tables

**Table 1:** Signalment, clinical signs, and laboratory parameters of the four cats with FIP-associated myocarditis before treatment (day 1), on day 42 of treatment, and at the end of treatment (day 84) with GS-441524.

Parameter	Study Patients Days After the Start of Treatment											
	**Cat 1**			**Cat 2**			**Cat 3**			**Cat 4**		
	**D1**	**D42**	**D84**	**D1**	**D42**	**D84**	**D1**	**D42**	**D84**	**D1**	**D42**	**D84**
**Signalment**												
Age (months)	70.10	71.60	73.10	48.10	49.60	51.10	5.70	7.20	8.70	104.20	105.70	107.20
Sex	male neutered			male neutered			male			female spayed		
Breed	Ragdoll			Domestic Shorthair			Holy Birman			Oriental Shorthair		
Weight (kg)	5.30	4.95	5.40	4.66	4.73	4.80	1.95	2.67	3.85	4.40	4.20	4.10
Clinical signs on D1	reduced general condition, hyporexia, fever			reduced general condition, hyporexia, fever			reduced general condition, hyporexia, fever			reduced general condition, hyporexia		
Neurological/ ocular signs	−/−			−/−			−/uveitis and chorioretinitis			−/−		
Location of effusion on D1	ascites, pleural effusion			ascites			ascites			ascites		
Volume of effusion on D1 *	+++			+++			+/−			++		
Ultrasonography findings on D1	severe volume of ascites, hyperechoic mesentery, lymphadenomegaly, hypoechoic pancreas			severe volume of ascites, hyperechoic mesentery, lymphadenomegaly, gallbladder wall edema			small volume of ascites, hyperechoic mesentery, lymphadenomegaly, hypoechoic pancreas, gallbladder wall edema, renomegaly			moderate volume of ascites, hyperechoic mesentery, lymphadenomegaly, hypoechoic pancreas, gallbladder wall edema, nephrolithiasis		
Lakhdhir criteria for diagnosis of myocarditis on D1	cTnI = 1.37 diagnosed FIP ^#^			cTnI = 1.20 diagnosed FIP ^#^			cTnI = 2.27 diagnosed FIP ^#^			cTnI = 5.84 diagnosed FIP ^#^		
**Complete blood cell count**												
Hematocrit (L/L) RI: 33–44	**29.20**	35.60	**31.60**	38.80	41.80	38.50	**22.90**	37.10	33.80	**26.70**	40.80	**47.20**
Platelet count (×10^9^/L) RI: 180–550	**36.0** ^§^	300.0	313.0	282.0	321.0	310.0	**21.0** ^§^	**113.0** ^¶^	274.0	**78.0** ^§^	219.0	200.0
White blood cell count (×10^9^/L) RI: 6–11	**16.94**	7.08	7.57	**13.74**	7.88	6.60	**2.72**	10.70	**11.13**	**18.45**	10.16	8.78
Neutrophil count (×10^9^/L) RI: 3–11.6	**15.87**	3.74	4.14	**12.84**	4.38	3.05	**1.33**	5.95	5.66	**16.79**	6.96	5.24
Lymphocyte count (×10^9^/L) RI: 1–4	**0.36**	2.65	1.94	**0.65**	2.43	2.37	1.25	3.08	**4.37**	**0.45**	2.36	2.66
Eosinophil count (×10^9^/L) RI: 0.04–0.6	0.06	0.41	**1.00**	**0.02**	**0.83**	**0.95**	0.05	**1.01**	**0.70**	**0.00**	0.39	0.44
Monocyte count (×10^9^/L) RI: 0.04–0.5	**0.65**	0.27	0.45	0.23	0.20	0.17	0.08	**0.65**	0.41	0.37	0.37	0.36
**Serum Biochemistry**												
Bilirubin (µmol/L) RI: 0–4.74	**15.20**	0.30	0.40	**24.60**	0.30	0.40	**12.60**	0.30	0.10	**71.50**	1.10	0.60
Total protein (g/L) RI: 60–85	**55.40**	67.70	63.30	78.90	75.50	67.50	**104.50**	67.30	66.90	**47.90**	73.40	68.20
Albumin (g/L) RI: 26–56	**20.40**	31.30	30.60	26.60	35.30	34.40	**21.90**	38.60	37.10	**16.50**	33.60	34.10
Globulins (g/L) RI: <55	35.00	36.40	32.70	52.30	40.20	33.10	**82.60**	28.70	29.80	31.40	39.80	34.10
A/G ratio RI: >0.6	**0.58**	0.86	0.94	**0.51**	0.88	1.04	**0.27**	1.34	1.24	**0.53**	0.84	1.00
SAA (mg/L) RI: <3.9	**46.00**	4.00	2.00	**96.70**	1.20	**5.70**	**76.20**	2.10	0.80	**77.90**	1.50	<0.30
AGP (µg/mL) RI: <567	**3924.4**	330.6	173.0	**3595.4**	226.1	191.1	**1854.4**	341.0	452.5	**4778.9**	315.7	271.0
**Viral Parameters**												
Blood viral load (copies/mL)	3625	0	0	0	0	0	182,012	0	0	726	0	0
Effusion viral load (copies/mL)	3.4 Mio.	− ^+^	− ^+^	95,381	− ^+^	− ^+^	28,073	− ^+^	− ^+^	3.7 Mio.	− ^+^	− ^+^
Fecal viral load (copies/g)	2.0 Mio.	0	0	2.9 Mio.	0	0	− ^+^	0	0	4561	0	0
Anti-FCoV antibody titers	1:400	1:400	1:400	1:400	1:400	1:400	1:1600	1:400	1:100	1:400	1:400	1:100

Values marked in bold are outside the reference intervals. * volume of effusion: +++ severe; ++ moderate, +/− small volume of effusion; ^#^ positive RT-qPCR out of effusion; ^§^ no platelet aggregates; ^¶^ platelet aggregates; ^+^ no sample. D1 = day 1; D84 = day 84; cTnI = cardiac troponin I; FIP = feline infectious peritonitis; RI = reference interval; A/G ratio = albumin/globulin ratio; SAA = serum amyloid A; AGP = alpha-1-acid-glycoprotein; FCoV = feline coronavirus.

**Table 2:** Additional symptomatic and cardiovascular treatment and other unrelated diseases developing during treatment with GS-441524.

Additional Treatment				
Initial symptomatic treatment ^+^	fluid therapy ^1^, amoxicillin/clavulanic acid (15 mg/kg, q8h, IV), mirtazapine, maropitant (1 mg/kg, q24h, IV), probiotics, buprenorphine (0.01 mg/kg, q8h, IV)	fluid therapy ^1^, mirtazapine, maropitant (1 mg/kg, q24h, IV)	fluid therapy ^1^, amoxicillin/clavulanic acid (15 mg/kg, q8h, IV), mirtazapine, maropitant (1 mg/kg, q24h, IV), ondansetron (0.2 mg/kg, q8h, IV), prednisolone acetate eye drops, atropine ointment	fluid therapy ^1^, amoxicillin/clavulanic acid (15 mg/kg, q8h, IV), mirtazapine, maropitant (1 mg/kg, q24h, IV), ondansetron (0.2 mg/kg, q8h, IV), terazosin (0.5 mg/cat, q24h, PO), fentanyl (6 µg/kg/h IV), methadone (0.2 mg/kg, q6h, IV)
Cardiovascular treatment after cardiac assessment	none	atenolol (1.5 mg/kg, q12h, PO)	furosemide (1.25 mg/kg, q12h, PO), pimobendan (0.3 mg/kg, q12h, PO), rivaroxaban (0.6 mg/kg, q24h, PO)	pimobendan (0.24 mg/kg, q12h, PO) clopidogrel (18.75 mg/cat, q24h, PO)
**Other unrelated diseases developing during treatment**				
	eye discharge and fever on day 35; trauma treated with meloxicam and antibiotics on day 62	none	reduced general condition, fever, vomiting, erythema cutis, sinus tachycardia with a single episode of VT on day 12	pyelectasis (treated with a subcutaneous ureteral bypass) on day 42

^+^ additional treatment was administered in parallel with antiviral treatment during hospitalization. ^1^ fluid therapy with Sterofundin with potassium supplementation at 20 mmol/L for dehydration at an individual dosage calculated by rehydration and maintenance needs. IV = intravenously, PO = per os, q24h = every 24 h, q12h = every 12 h, q8h = every 8 h, q6h = every 6 h, VT = ventricular tachycardia.

**Table 3:** Echocardiographic measurements of the two equivocal cats (cats 38 and 40) before treatment (day 1), on day 42 of treatment, and at the end of treatment (day 84) with GS-441524.

Values		Before Treatment		Day 42		Day 84	
	**RI**	**C38**	**C40**	**C38**	**C40**	**C38**	**C40**
IVSd	<5.00	5.30	5.10	4.80	4.90	4.70	4.30
LVIDd	13.40–20.90	15.70	16.60	17.00	17.60	15.80	18.20
LVPWd	<5.00	5.40	5.60	4.90	4.80	4.90	4.90
LVIDs	6.00–13.70	10.40	9.70	10.50	11.70	9.30	11.10
LA/Ao	<1.60	1.22	1.32	1.44	1.44	1.30	1.18
LAD	<16.00	14.00	15.00	13.00	14.00	13.00	13.00
RAD	<12.00	11.20	10.90	9.70	11.50	11.80	11.70
GLS	>−21.18	−23.71	−22.19	−23.67	−25.15	−24.97	−28.77
cTnI	<0.06	1.37	1.20	– *	− *	− *	−*

* no sample. C38 = cat 38; C40 = cat 40; IVSd = interventricular septum thickness at end-diastole in mm; LVIDd = left ventricular internal diameter at end-diastole in mm; LVPWd = left ventricular posterior wall thickness at end-diastole in mm; LVIDs = left ventricular internal diameter at end-systole in mm; LA/Ao = left atrium-to-aortic root ratio; LAD = left atrial diameter in mm; RAD = right atrial diameter in mm; RI = reference interval; GLS = global longitudinal strain in %; cTnI = cardiac troponin I in ng/mL.

**Table 4:** Echocardiographic measurements of two cats with systolic dysfunction (cats 24 and 39) before treatment (day 1), on day 42 of treatment and at the end of treatment (day 84) with GS-441524.

Values		Before Treatment		Day 42		Day 84	
	**RI**	**C24**	**C39**	**C24**	**C39**	**C24**	**C39**
IVSd	<5.00	2.80	3.60	3.70	3.60	3.70	3.50
LVIDd	11.40–17.80 (C24) 12.20–19.20 (C39)	16.00	16.40	17.00	16.80	14.50	16.40
LVPWd	<5.00	3.70	4.50	3.80	4.00	4.60	4.10
LVIDs	5.10–11.70 (C24) 5.50–12.60 (C39)	13.50	13.10	12.90	12.40	10.60	8.90
LA/Ao	<1.60	2.43	1.75	1.75	1.38	1.42	1.44
LAD	<16.00	19.00	17.00	15.00	14.00	14.00	13.00
RAD	<12.00	13.10	14.90	11.50	11.50	10.90	10.70
GLS	>−21.18	−10.19	−16.22	−17.22	−24.64	−28.29	−30.34
cTnI	<0.06	2.27	5.84	– *	– *	– *	– *

* no sample. C24 = cat 24; C39 = cat 39; IVSd = interventricular septum thickness at end-diastole in mm; LVIDd = left ventricular internal diameter at end-diastole in mm; LVPWd = left ventricular posterior wall thickness at end-diastole in mm; LVIDs = left ventricular internal diameter at end-systole in mm; LA/Ao = left atrium-to-aortic root ratio; LAD = left atrial diameter in mm; RAD = right atrial diameter in mm; RI = reference interval; GLS = global longitudinal strain in %; cTnI = cardiac troponin I in ng/mL.

## Data Availability

The authors confirm that the datasets analyzed during the study are available from the corresponding author upon reasonable request.

## Abbreviations

The following abbreviations are used in this manuscript:

LMU	Ludwig Maximilians University
FIP	Feline infectious peritonitis
FCoV	Feline coronavirus
cTnI	Cardiac Troponin I
ECG	Electrocardiography
FIV	Feline immunodeficiency virus
HCM	Hypertrophic cardiomyopathy
SARS-CoV-2	Severe acute respiratory syndrome coronavirus 2
IHC	Immunohistochemistry
DCM	Dilated cardiomyopathy
FeLV	Feline leukemia virus
RT-qPCR	Reverse transcription quantitative polymerase chain reaction
A/G	Albumin/globulin ratio
q24h	Every 24 h
PO	Per os
CBC	Complete blood cell count
RI	Reference interval
VPCs	Ventricular premature contractions
APCs	Atrial premature contractions
VT	Ventricular tachycardia
IVSd	Interventricular septal thickness in end-diastole
LVPWd	Left ventricular posterior wall thickness in end-diastole
LV	Left ventricular
LA/Ao	Left atrial-to-aortic root ratio
LAD	Left atrial diameter
RAD	Right atrial diameter
LVIDs	Left ventricular internal diameter in end-systole
LVIDd	Left ventricular internal diameter in end-systole
GLS	Global longitudinal strain
CHF	Congestive heart failure
q8h	Every 8 h
q12h	Every 12 h
q6h	Every 6 h
COVID-19	Coronavirus disease 2019
TMT	Transient myocardial thickening
UK	United Kingdom
